# Remote consent approaches for mobile phone surveys of non-communicable disease risk factors in Colombia and Uganda: A randomized study

**DOI:** 10.1371/journal.pone.0279236

**Published:** 2022-12-21

**Authors:** Joseph Ali, Madhuram Nagarajan, Erisa S. Mwaka, Elizeus Rutebemberwa, Andres I. Vecino-Ortiz, Angelica Tórres Quintero, Mariana Rodriguez-Patarroyo, Vidhi Maniar, Gulam Muhammed Al Kibria, Alain B. Labrique, George W. Pariyo, Dustin G. Gibson

**Affiliations:** 1 Department of International Health, Johns Hopkins Bloomberg School of Public Health, Baltimore, Maryland, United States of America; 2 Berman Institute of Bioethics, Johns Hopkins University, Baltimore, Maryland, United States of America; 3 College of Health Sciences, Makerere University, Kampala, Uganda; 4 Public Health Institute, Pontificia Universidad Javeriana, Bogotá, Colombia; 5 Bioethics Institute, Pontificia Universidad Javeriana, Bogotá, Colombia; University of Kurdistan Hewler, IRAQ

## Abstract

**Introduction:**

Automated mobile phone surveys (MPS) can be used to collect public health data of various types to inform health policy and programs globally. One challenge in administering MPS is identification of an appropriate and effective participant consent process. This study investigated the impact of different survey consent approaches on participant disposition (response characteristics and understanding of the purpose of the survey) within the context of an MPS that measured noncommunicable disease (NCD) risk factors across Colombia and Uganda.

**Methods:**

Participants were randomized to one of five consent approaches, with consent modules varying by the consent disclosure and mode of authorization. The control arm consisted of a standard consent disclosure and a combined opt-in/opt-out mode of authorization. The other four arms consist of a modified consent disclosure and one of four different forms of authorization (i.e., opt-in, opt-out, combined opt-in/opt-out, or implied). Data related to respondent disposition and respondent understanding of the survey purpose were analyzed.

**Results:**

Among 1889 completed surveys in Colombia, differences in contact, response, refusal, and cooperation rates by study arms were found. About 68% of respondents correctly identified the survey purpose, with no significant difference by study arm. Participants reporting higher levels of education and urban residency were more likely to identify the purpose correctly. Participants were also more likely to accurately identify the survey purpose after completing several survey modules, compared to immediately following the consent disclosure (78.8% vs 54.2% correct, p<0.001). In Uganda, 1890 completed surveys were collected. Though there were differences in contact, refusal, and cooperation rates by study arm, response rates were similar across arms. About 37% of respondents identified the survey purpose correctly, with no difference by arm. Those with higher levels of education and who completed the survey in English were able to more accurately identify the survey purpose. Again, participants were more likely to accurately identify the purpose of the survey after completing several NCD modules, compared to immediately following the consent module (42.0% vs 32.2% correct, p = 0.013).

**Conclusion:**

This study contributes to the limited available evidence regarding consent procedures for automated MPS. Future studies should develop and trial additional interventions to enhance consent for automated public health surveys, and measure other dimensions of participant engagement and understanding.

## Introduction

As has been well documented, low- and middle-income countries (LMICs) are experiencing major shifts in disease burdens, with significant increases in the prevalence of noncommunicable diseases (NCDs) [1]. The paucity and cost of acquiring timely data on the underlying prevalence of NCDs in many countries makes the impact of any public health intervention difficult to evaluate, especially where target populations are relatively hard to reach [2, 3]. Evidence-based policy development to advance NCD control is a key goal, and effective approaches need to be advanced if countries are to meet local and global targets [4].

Given the widespread access to mobile phones globally and rapidly expanding capabilities of digital devices, mobile technology is increasingly finding a place in monitoring communicable and noncommunicable diseases, and their associated risk factors [5–7]. Simple mobile phone surveys (MPS) deployed through live operator administered interviews, pre-recorded automated interactive voice response (IVR) surveys, or automated text messaging have recently been trialed to facilitate large-scale population monitoring of NCD risk factors in Colombia, Uganda, and other countries [8–12]. What makes these efforts particularly attractive to policymakers, public health practitioners, and researchers is their relatively low cost, minimal technological requirements, and potential to cut data collection and processing times down from year(s) to weeks [5, 7].

While mobile phone-based NCD risk factor monitoring presents opportunities for the advancement of global public health surveillance, emerging practices ought to align with and inform the evolution of existing norms and requirements for ethics and governance of public health surveillance and research [9, 12, 13]. Unfortunately, little empirical evidence exists to advance thoughtful application of these requirements to the novel terrain of MPS. Studies are needed to advance understanding of a variety of data-related considerations–e.g., data privacy, security, ownership, access and use–and also to support careful development and implementation of consent processes that promote adequate disclosure, sufficient depth of comprehension, and authentic remote authorization during MPS [12, 14–16]. Systematic assessment of these and other ethics and regulatory considerations is critical to the stewardship of a foundation of trust upon which much of global digital health rests.

Recent qualitative and conceptual research related to consent for MPS suggests a challenge with identifying and prioritizing key information during very concise consent disclosures, a potential for inadequate respondent understanding, and a risk of uninformed “click-through” authorization with remote consent and automated disclosure processes [9, 12, 17]. Remote consent is defined as a consent process with no in-person components, [18] while automated disclosure refers to use of only pre-recorded informational messages during the process of consent. Experimental research focused on optimization of consent processes for large-scale MPS to monitor disease risk factors is needed to advance remote and automated forms of consent in digital health. Such empirical efforts can build on and complement the relatively large existing body of interventional consent research, which has been conducted primarily in non-digital health contexts [14–16].

In order to advance empirical understanding of consent for remote and automated health surveys in LMICs, we conducted a randomized study comparing different approaches to the consent process within a large automated IVR of NCD risk factors in two countries, Colombia and Uganda. The present study was implemented in both countries because of previous and ongoing collaborative MPS efforts in each targeting NCD risk factors, and to explore our research questions in two sociodemographic, culturally, and geographically distinct contexts. We comparatively tested the impacts of modified consent approaches on respondent disposition (e.g., response and completion rates) and participants’ understanding of the purpose of the mobile phone survey–a simple but important indicator of basic survey understanding.

## Methods

### Study design

We conducted a randomized controlled multifactor trial that tested variations to the consent process for a MPS designed to collect self-reported NCD risk factor data. The risk factors studied related to tobacco use, alcohol intake, diet, physical activity, and accessing screening for selected NCDs. We investigated the influence of i) the disclosure language used to convey survey consent information, and ii) the mode of authorization (i.e., opt-in, opt-out, combined opt-in/opt-out, or implied) on respondents, primarily by measuring response and cooperation rates as well as participant understanding of the purpose of the survey.

**Fig 1:**
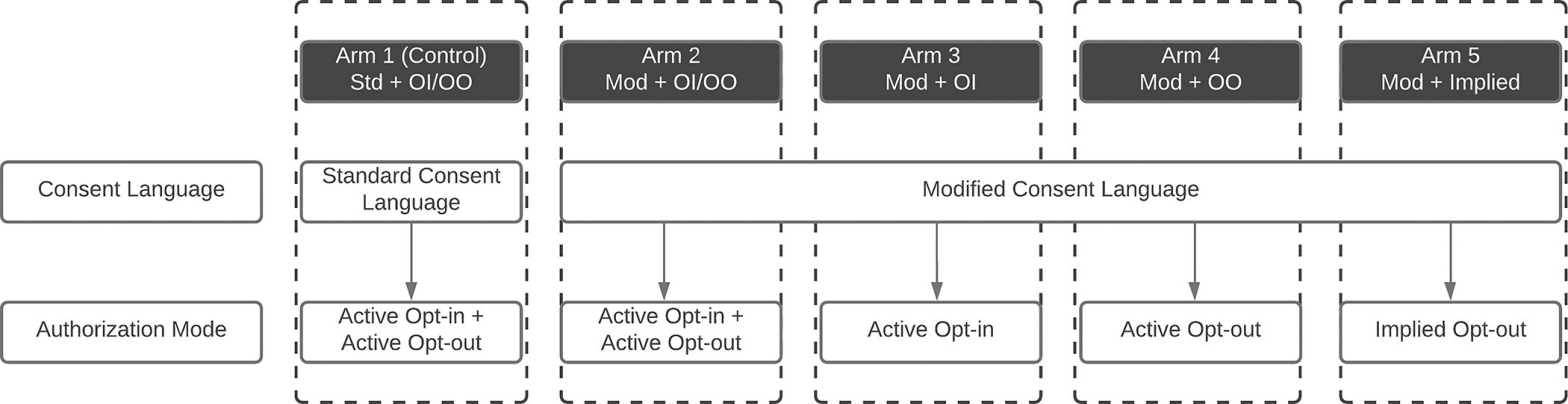
Study design and arm labels.

Participants were randomized to one of five study arms. In each country, the control arm consisted of a standard consent disclosure and a combined opt-in/opt-out mode of authorization, as was developed for a parent study [8]. The other four arms consisted of a modified consent disclosure and one of the four different modes of authorization described above (**Fig 1**, also described in the Interventions section).

The study was registered with ClinicalTrials.gov [19] and received ethics clearance from the Johns Hopkins University Bloomberg School of Public Health, USA; Public Health Institute of Pontificia Universidad Javeriana, Colombia; Makerere University School of Public Health, Uganda; and the Uganda National Council for Science and Technology, Uganda.

### Interventions

The first type of intervention was a modification to the language used to disclose information about the survey to potential respondents. In order to develop the modified disclosures, standard consent disclosure language used in the parent study [8] was augmented following a formative, qualitative phase to elicit consent preferences and expectations of mHealth researchers, ethicists, and members of the public in Colombia and Uganda [12, 20, 21]. Wording of standard and modified disclosures is provided in **S1 Table**.

The second intervention was to randomly vary the way in which respondents were requested to signal their interest or refusal to complete a survey using their mobile phone keypad. Variations on modes of authorization included both active and passive modalities and were: (i) opting-in (“press 1 if you would like to complete the survey”); (ii) opting-out (“press 3 if you do not want to complete the survey”); (iii) a combination of opting-in and opting-out (“press 1 if you would like to complete the survey or 3 if you no not want to complete it”); and (iv) implied authorization (“by completing this survey you agree to participate”) where participants did not have to press a keypad button. The control arm was ‘standard introduction along with opt in and opt out’ while other four arms were the combinations of modified into with any of the four above-mentioned modes of authorization (S1 Table). Potential participants who were age-eligible were provided an audio consent disclosure statement and were requested to authorize their participation as specified for each study arm. We also tested the timing of asking the survey purpose understanding question, with some respondents randomized to receive the survey item early in the survey and others near the end of the survey (S2 Table). In all instances, a potential respondent could refuse a survey by not picking up the phone or by hanging up.

### Participants

**Fig 2:**
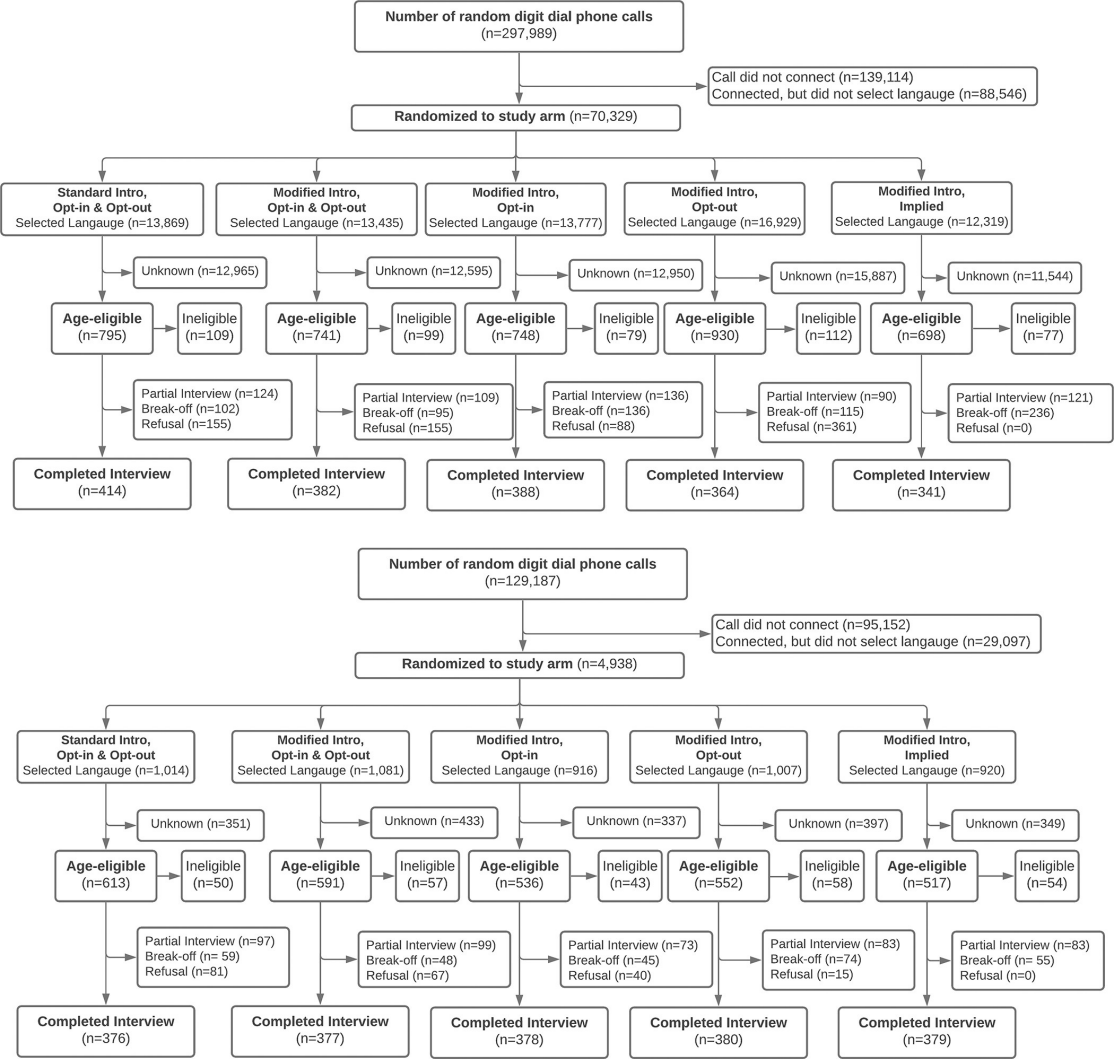
**a.** CONSORT diagram of interview outcomes among participants in Colombia. **b.** CONSORT diagram of interview outcomes among participants in Uganda.

Study participants were sampled through the use of random digit dialing (RDD) [8]. A pseudo-random number generator was used to produce random numbers of seven-digit length, prefixed with (randomly generated) mobile network operator-specific three-digit phone codes and country codes for Colombia (57) and Uganda (256). The RDD was executed through the IVR platform. In Colombia, 297,989 RDD calls were made from 14 November 2018 until 25 January 2019, resulting in 1,889 completed surveys across all five arms **(Fig 2A)**. Between 31 August 2018 and 1 October 2018, a total of 129,187 calls were made in Uganda, with 1,890 completed surveys across all arms **(Fig 2B)**.

Individuals who received a phone call from the IVR system were screened for age-based eligibility (i.e., ≥18 years-old). Age-eligibility was determined based on self-report; participants were instructed to use the keypad to indicate their age *(i*.*e*., *“Are you 18 years or older*? *If YES*, *Press 1*. *If No*, *Press 3”)*. Age-eligible participants were provided with information about the survey and invited to participate. Eligibility- and consent-screened participants were then presented with the remainder of the IVR survey.

### Randomization and masking

Randomization was done in the IVR platform in a 1:1:1:1:1 allocation ratio across the five study arms. Participants were automatically randomized to different study arms after choosing a preferred survey language and were unaware of their study group allocation. Data cleaning was done by researchers blinded to the allocation of study participants; analyses were conducted unblinded.

### Study procedures and instruments

The study procedures for Colombia and Uganda were comparable but were operationalized using different available IVR survey delivery platforms, which introduced minor variations in delivery. Specific differences in procedure are discussed where applicable.

Participants who picked up a phone call were asked to indicate their language preference through a numeric response on the keypad. In Colombia, the survey was administered only in Spanish; in Uganda, the languages available were Luganda, Luo, Runyakitara, and English. In both countries, the languages offered were commonly understood across a large portion of the population. Survey questions were administered through delivery of several modules: i) language selection (as applicable), ii) survey introduction, iii) screening and authorization, iv) demographics, v) NCD risk factors (five modules), and vi) survey understanding measure. The order of administration of the different NCD modules was randomized to minimize bias due to mid-survey drop-off, while keeping the question order within modules constant and preserving skip patterns. The survey understanding measure was also randomly assigned to one of six positions within the survey in Uganda, and to either the beginning or end of the NCD module in Colombia. This variation in assignment across countries reflected the different capabilities of the survey delivery platforms.

The items in each NCD module were based on standardized questions from widely used surveys, chosen after expert review and adapted to the country context through multiple rounds of testing and feedback. The survey understanding item was adapted from previous consent understanding studies [22–24], with some country-specific variation to accommodate local linguistic (semantic and syntactic) requirements (**S2 Table**). Translated text was recorded, back-translated, and audio files of the questionnaire were tested at country-level prior to survey deployment to ensure that the translations and recordings were comprehensible.

The IVR surveys were deployed between 08:00 AM and 08:00 PM local time, and a single contact attempt made with each randomly generated number. Survey participants could choose to repeat questions through key presses on their mobile phones as they moved through the survey. Participants did not incur charges for the airtime taken to complete the survey and were informed of this; those who completed the IVR survey received a small airtime incentive delivered through their cellular provider.

### Outcomes

The study had three primary outcomes: response rate, cooperation rate, and understanding of the purpose of the survey. Secondary outcomes were contact rate and refusal rate. Disposition-related survey outcomes were categorized using standard definitions from the American Association for Public Opinion Research (AAPOR). The calculation method (i.e., numerators, denominators, and equations) of contact, response, refusal, and cooperation rates are presented in **S3 Table** [25]. To capture respondent understanding of the survey purpose, participants had to select one of the following four categories, indicating what they perceived as the purpose: to improve understanding of hospital services, to improve understanding of community health, to develop a new medicine, or don’t know. We considered ‘to improve community health’ as the correct response.

### Statistical analysis

We used identical assumptions to calculate the required sample size in both countries. To produce the largest sample size with adequate power, the following assumptions were used: cooperation rate of 30%, a type I error (i.e., alpha) of 0.05, and 80% power. To detect a minimum absolute difference of 10%, a total of 376 participants were required in each arm in each country. We did not inflate sample sizes for multiple comparisons, as recommended in Rothman [26].

The survey introduction, different modes of authorization, and timing of measurement were the independent variables. Participation rates (i.e., contact, response, refusal, and cooperation rates) and understanding of the survey purpose were the dependent variables. First, demographic characteristics of complete interviews were described using numbers and percentages (%) for both countries. Then, log-binomial regression was used to calculate the risk ratios (RR) and corresponding 95% confidence intervals (CI) for contact, response, refusal, and cooperation rates by study arms. Proportions of survey understanding were compared across demographics and study arms using chi-squared tests. The effect of measurement timing on understanding of the survey’s purpose–measurement immediately following information disclosure vs later in the survey–was also explored. Lastly, unadjusted and adjusted log-binomial regression analyses were used to report the RR (with 95% CI) for the likelihood of the association of correct response with survey arms and sociodemographic characteristics. The adjustments were done for the following sociodemographic variables: age, gender, education, and rural-urban location of residence. The log-binomial regression shows significance level using Z-test. We did the adjustments to account for sociodemographic conditions as they may confound the association of outcomes and interventions of the study. Analyses were done with Stata/SE (version 14.1; Stata Corp, College Station, TX, USA) [27]. An alpha of 0.05 was assumed for all tests of statistical significance.

### Inclusivity in global research

Additional information regarding the ethical, cultural, and scientific considerations specific to inclusivity in global research is included in the (S1 Checklist).

## Results

### Survey and respondent characteristics

Demographic and socioeconomic characteristics of the completed surveys were similarly distributed across study arms **(Table 1).** There was a roughly even distribution between male and female participants, and about half of participants were between 18–29 years old. One-third of respondents had completed secondary education, one-fourth had completed a technical education, and one-third had completed tertiary education or higher. Three-fourths of the sample consisted of urban respondents, and the survey was conducted exclusively in Spanish.

**Table 1:** Demographics of complete interviews by study arm in Colombia and Uganda, n (%).

Demographics	Colombia					Uganda				
	Arm 1: SI, OI OO	Arm 2: MI, OI OO	Arm 3: MI, OI	Arm 4: MI, OO	Arm 5: MI, Implied	Arm 1: SI, OI OO	Arm 2: MI, OI OO	Arm 3: MI, OI	Arm 4: MI, OO	Arm 5: MI, Implied
	(n = 414)	(n = 382)	(n = 388)	(n = 364)	(n = 341)	(n = 376)	(n = 377)	(n = 378)	(n = 380)	(n = 379)
**Sex**										
Male	209 (50.5)	184 (48.2)	173 (44.6)	183 (50.3)	168 (49.3)	74 (19.7)	60 (15.9)	89 (23.5)	78 (20.5)	71 (18.7)
Female	205 (49.5)	198 (51.8)	215 (55.4)	181 (49.7)	173 (50.7)	302 (80.3)	317 (84.1)	289 (76.5)	302 (79.5)	308 (81.3)
**Age (years)**										
18–29	195 (47.1)	182 (47.6)	180 (46.4)	168 (46.2)	162 (47.5)	252 (67)	262 (69.5)	251 (66.4)	267 (70.3)	256 (67.6)
30–49	165 (39.9)	130 (34.0)	142 (36.6)	149 (40.9)	122 (35.8)	113 (30.1)	97 (25.7)	112 (29.6)	100 (26.3)	111 (29.3)
50+	54 (13.0)	70 (18.3)	66 (17.0)	47 (12.9)	57 (16.7)	11 (2.9)	18 (4.8)	15 (4)	13 (3.4)	12 (3.2)
**Education**										
None	9 (2.2)	2 (0.5)	5 (1.3)	3 (0.8)	4 (1.2)	55 (14.6)	54 (14.3)	56 (14.8)	50 (13.2)	53 (14)
Primary	50 (12.1)	61 (16.0)	53 (13.7)	42 (11.5)	35 (10.3)	102 (27.1)	99 (26.3)	86 (22.8)	109 (28.7)	107 (28.2)
Secondary	115 (27.8)	94 (24.6)	85 (21.9)	95 (26.1)	92 (27)	147 (39.1)	143 (37.9)	168 (44.4)	151 (39.7)	144 (38)
Technical	103 (24.9)	88 (23.0)	115 (29.6)	93 (25.6)	84 (24.6)	-	-	-	-	-
Tertiary/ higher	136 (32.9)	137 (35.9)	130 (33.5)	130 (35.7)	124 (36.4)	71 (18.9)	79 (21)	68 (18)	70 (18.4)	75 (19.8)
**Location**			** **	** **	** **					
Urban	307 (74.9)	284 (74.5)	272 (70.7)	262 (72.8)	255 (75.2)	184 (48.9)	189 (50.3)	179 (47.5)	195 (51.5)	192 (50.7)
Rural	103 (25.1)	97 (25.5)	113 (29.3)	98 (27.2)	84 (24.8)	192 (51.1)	187 (49.7)	198 (52.5)	184 (48.5)	187 (49.3)
**Language**										
Spanish	414 (100)	382 (100)	388 (100)	364 (100)	341 (100)	-	-	-	-	-
Luganda	-	-	-	-	-	198 (52.7)	209 (55.4)	213 (56.4)	209 (55)	187 (49.3)
Luo	-	-	-	-	-	33 (8.8)	30 (8)	20 (5.3)	32 (8.4)	37 (9.8)
Runyakitara	-	-	-	-	-	93 (24.7)	80 (21.2)	93 (24.6)	79 (20.8)	86 (22.7)
English	-	-	-	-	-	52 (13.8)	58 (15.4)	52 (13.8)	60 (15.8)	69 (18.2)

**Abbreviations:** MI: Modified Intro, NA: Not available, OI: Opt-in, OO: Opt-out, SI: Standard Intro

Demographic and socioeconomic characteristics of the completed surveys were similarly distributed across the study arms (**Table 1)**. Over three-fourths of the sample was female, and about two-thirds were between 18–29 years old. One-fourth had completed only primary education, and about 40% had completed secondary education. There was almost equal representation of rural and urban dwellers. Over half completed the survey in Luganda and about one-fourth in Runyakitara, with the remainder in English and Luo.

### Survey disposition according to study arm

In Colombia, the cooperation rates were 52.08% in the control arm (Arm 1), 51.55% in Arm 2, 51.87% in Arm 3, 39.14% in Arm 4, and 48.85% in Arm 5 **(Fig 1, Table 2)**. Cooperation rates were only significantly lower in Arm 4 (RR 0.75, 95% CI: 0.68–0.83, p<0.001) as compared to the control arm. The response rates were 1.09% in the control arm, 0.92% in Arm 2, 0.98% in Arm 3, 0.68% in Arm 4, and 0.93% in Arm 5. The response rates were significantly lower in Arm 2 (RR 0.85, 95% CI: 0.75–0.96, p = 0.008), Arm 4 (RR 0.62, 95% CI: 0.55–0.70, p<0.001), and Arm 5 (RR 0.86, 95% CI: 0.76–0.97, p = 0.02) when compared to the control arm. For secondary outcomes, the refusal rates were significantly lower in Arm 3 0.42% (RR, 0.81 95% CI: 0.68–0.97, p = 0.019) and significantly higher in Arm 4, 0.71% (RR 1.37, 95% CI: 1.17–1.59, p<0.001), in comparison to the control arm. Arms 2, 3, 4, and 5 had significantly lower contact rates than the control arm.

**Table 2:** Survey rates and risk ratio by study arm in Colombia and Uganda.

Survey Rate	Colombia					Uganda				
	Arm 1: SI, OI OO	Arm 2: MI, OI OO	Arm 3: MI, OI	Arm 4: MI, OO	Arm 5: MI, Implied	Arm 1: SI, OI OO	Arm 2: MI, OI OO	Arm 3: MI, OI	Arm 4: MI, OO	Arm 5: MI, Implied
**Contact Rate #2**	1.61%	1.39%	1.40%	1.39%	1.41%	2.59%	2.49%	2.27%	2.33%	2.19%
RR (95% CI)	*Ref*.	0.87 (0.78–0.96)	0.87 (0.79–0.96)	0.86 (0.79–0.95)	0.88 (0.79–0.97)	*Ref*.	0.96 (0.86–1.08)	0.88 (0.78–0.98)	0.90 (0.80–1.01)	0.85 (0.75–0.95)
p-value	*Ref*.	**0.005**	**0.007**	**0.002**	**0.011**	*Ref*.	0.49	**0.026**	0.073	**0.005**
**Response Rate #4**	1.09%	0.92%	0.98%	0.68%	0.93%	2.00%	2.01%	1.91%	1.96%	1.96%
RR (95% CI)	*Ref*.	0.85 (0.75–0.96)	0.90 (0.80–1.02)	0.62 (0.55–0.70)	0.86 (0.76–0.97)	*Ref*.	1.00 (0.89–1.14)	0.96 (0.84–1.09)	0.98 (0.86–1.11)	0.98 (0.86–1.11)
p-value	*Ref*.	**0.008**	0.093	**<0.001**	**0.016**	*Ref*.	0.95	0.50	0.75	0.76
**Refusal Rate #2**	0.52%	0.47%	0.42%	0.71%	0.48%	0.59%	0.48%	0.36%	0.38%	0.23%
RR (95% CI)	*Ref*.	0.90 (0.76, 1.08)	0.81 (0.68–0.97)	1.37 (1.17–1.59)	0.92 (0.77–1.09)	*Ref*.	0.82 (0.64–1.05)	0.61 (0.47–0.80)	0.64 (0.49–0.83)	0.39 (0.29–0.54)
p-value	*Ref*.	0.26	**0.019**	**<0.001**	0.34	*Ref*.	0.11	**<0.001**	**0.001**	**<0.001**
**Cooperation Rate #1**	52.08%	51.55%	51.87%	39.14%	48.85%	61.34%	63.79%	70.52%	68.84%	73.31%
RR (95% CI)	*Ref*.	0.99 (0.90–1.09)	1.00 (0.90–1.10)	0.75 (0.68–0.83)	0.94 (0.85–1.04)	*Ref*.	1.04 (0.95–1.13)	1.15 (1.06–1.25)	1.12 (1.03–1.22)	1.20 (1.10–1.30)
p-value	*Ref*.	0.84	0.94	**<0.001**	0.22	*Ref*.	0.38	**0.001**	**0.007**	**<0.001**

**Abbreviations:** CI: Confidence interval, MI: Modified Intro, OI: Opt-in, OO: Opt-out, SI: Standard Intro

In Uganda, the cooperation rates were 61.34% in the control arm, 63.79% in Arm 2, 70.52% in Arm 3, 68.84% in Arm 4, and 73.31% in Arm 5 **(Fig 1, Table 2)**. Cooperation rates were significantly higher in Arm 3 (RR 1.15, 95% CI: 1.06–1.25, p = 0.001), Arm 4 (RR 1.12, 95% CI: 1.03–1.22, p = 0.007), and Arm 5 (RR 1.20, 95% CI: 1.10–1.30, p<0.001) as compared to the control arm. There was no significant difference in response rates across the different study arms. The refusal rates were significantly lower in Arm 3 (0.36%, RR 0.61, 95% CI: 0.47–0.8, p<0.001), Arm 4 (0.38%, RR 0.64, 95% CI: 0.49–0.83, p = 0.001), and Arm 5 (0.23%, RR 0.39, 95% CI: 0.29–0.54, p<0.001) in comparison to the control arm. Arms 3 and 5 had significantly lower contact rates than the control arm.

We also reported the differences in outcomes using Arm 2 as the reference category **(Table 3)**. In Colombia, the response rate (RR: 0.73, 95% CI: 0.65–0.83, p <0.001), cooperation rate (RR: 0.76, 95% CI: 0.68–0.84, p <0.001) were only lower in Arm 4; however, this arm had higher refusal rate (RR: 1.51, 95% CI: 1.30–1.76, p <0.001).

**Table 3:** Survey rates and risk ratios among the modified consent arms with the modified opt-out opt-in arm as reference in Colombia and Uganda.

	Colombia					Uganda				
	Arm 1: SI, OI OO	Arm 2: MI, OI OO	Arm 3: MI, OI	Arm 4: MI, OO	Arm 5: MI, Implied	Arm 1: SI, OI OO	Arm 2: MI, OI OO	Arm 3: MI, OI	Arm 4: MI, OO	Arm 5: MI, Implied
**Contact Rate #2**	1.61%	1.39%	1.40%	1.39%	1.41%	2.59%	2.49%	2.27%	2.33%	2.19%
RR (95% CI)	1.15 (1.04–1.28)	*Ref*.	1.01 (0.91–1.11)	1.00 (0.91–1.10)	1.01 (0.91–1.12)	1.04 (0.93–1.16)	*Ref*.	0.91 (0.81–1.02)	0.94 (0.84–1.05)	0.88 (0.78–0.99)
p-value	**0.005**	*Ref*.	0.91	0.94	0.81	0.50	*Ref*.	0.12	0.27	**0.032**
**Response Rate #4**	1.09%	0.92%	0.98%	0.68%	0.93%	2.00%	2.01%	1.91%	1.96%	1.96%
RR (95% CI)	1.17 (1.04–1.33)	*Ref*.	1.06 (0.94–1.20)	0.73 (0.65–0.83)	1.01 (0.89–1.15)	1.00 (0.88–1.13)	*Ref*.	0.95 (0.84–1.08)	0.98 (0.86–1.11)	0.98 (0.86–1.11)
p-value	**0.008**	*Ref*.	0.33	**<0.001**	0.86	0.95	*Ref*.	0.46	0.70	0.72
**Refusal Rate #2**	0.52%	0.47%	0.42%	0.71%	0.48%	0.59%	0.48%	0.36%	0.38%	0.23%
RR (95% CI)	1.11 (0.93–1.31)	*Ref*.	0.89 (0.75–1.07)	1.51 (1.30–1.76)	1.02 (0.85–1.21)	1.22 (0.95–1.56)	*Ref*.	0.74 (0.56–0.98)	0.78 (0.59–1.02)	0.48 (0.35–0.66)
p-value	0.26	*Ref*.	0.22	**<0.001**	0.87	0.11	*Ref*.	**0.038**	0.072	**<0.001**
**Cooperation Rate #1**	52.08%	51.55%	51.87%	39.14%	48.85%	61.34%	63.79%	70.52%	68.84%	73.31%
RR (95% CI)	1.01 (0.92–1.11)	*Ref*.	1.01 (0.91–1.11)	0.76 (0.68–0.84)	0.95 (0.85–1.05)	0.96 (0.88–1.05)	*Ref*.	1.11 (1.02–1.20)	1.08 (0.99–1.17)	1.15 (1.06–1.24)
p-value	0.84	*Ref*.	0.90	**<0.001**	0.31	0.38	*Ref*.	**0.016**	0.071	**0.001**

**Abbreviations:** CI: Confidence interval, MI: Modified Intro, NA: Not available, OI: Opt-in, OO: Opt-out, RR: Risk ratio, SI: Standard Intro

In Uganda, contact rate was only significantly lower on Arm 5 (RR: 0.88, 95% CI: 0.78–0.99, p = 0.032) **(Table 3)**. The response rate did not differ by arm. Refusal rate was significantly lower in Arm 3 (RR: 0.74, 95% CI: 0.56–0.98, p = 0.038) and Arm 5 (RR: 0.48, 95% CI: 0.56–0.98, p<0.001). These two arms also had significantly higher cooperation rates.

Disposition codes by intervention arms for both countries are also reported in full in **S4 Table**.

### Respondent understanding of survey purpose

**Fig 3:**
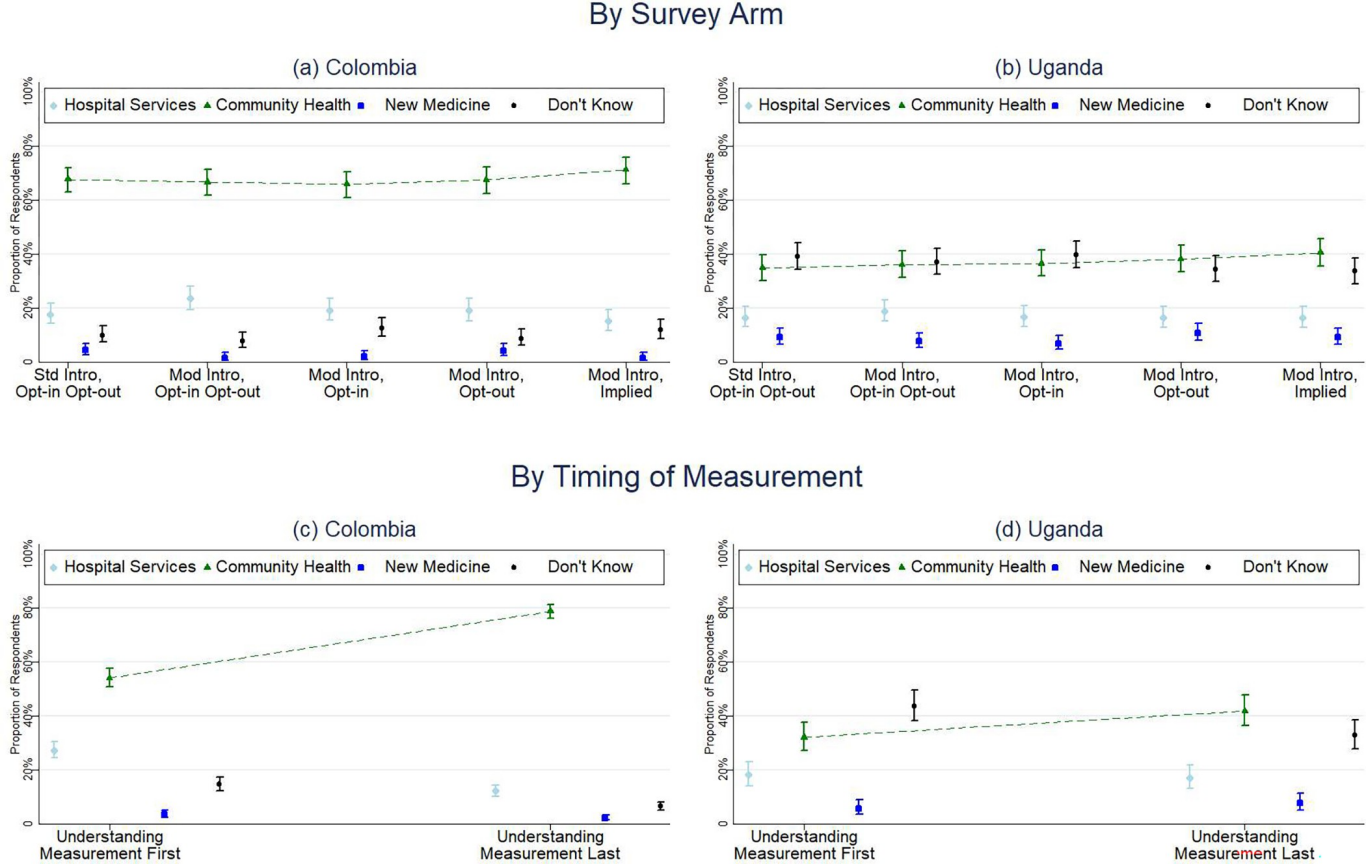
Understanding of survey purpose by study arm and timing of measurement in Colombia and Uganda.

**Fig 4:**
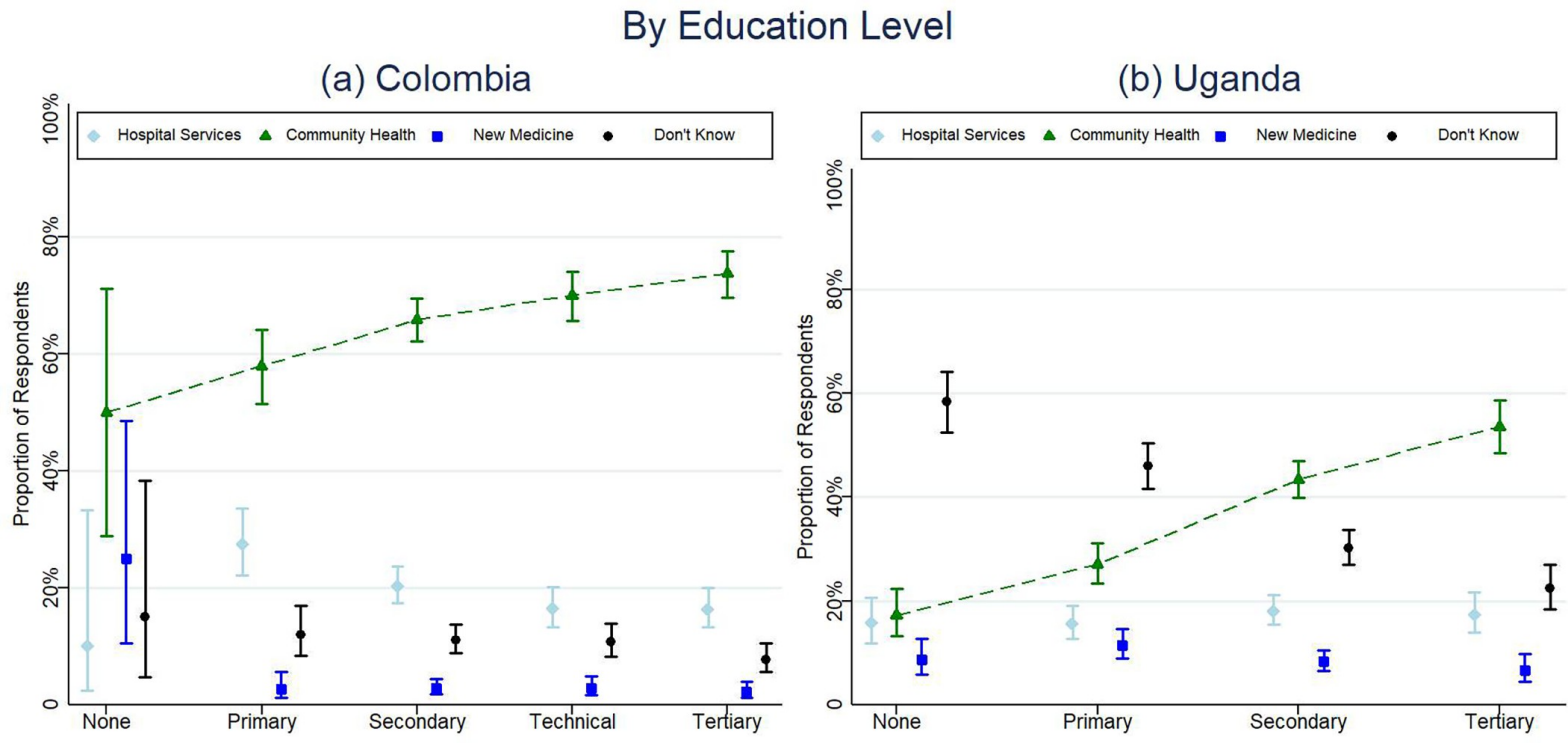
Understanding by participant’s education in Colombia and Uganda.

In Colombia, overall, 67.82% of survey respondents correctly identified the purpose of the survey (95% CI: 65.64–69.93), with no significant difference by study arm, age, or sex **(Table 4, Fig 3A)**. The proportion that correctly identified the survey purpose increased with educational attainment (50.00% none, 57.93% primary, 65.88% secondary, 70.02% technical, 73.76% tertiary, p<0.001) **(Table 4, Fig 4A)**. A significantly higher proportion of urban respondents (p = 0.003) correctly identified the survey purpose (69.24%), compared to rural respondents (63.90%). A significantly higher proportion (p<0.001) of survey respondents correctly identified the survey purpose after having completed a majority of the survey (78.84%, 95% CI: 76.20–81.26) as compared to respondents who were asked about the survey purpose shortly after consent (54.24%, 95% CI: 50.79–57.64) **(Fig 3C)**.

**Table 4:** Survey understanding by demographics in Colombia and Uganda, n (%).

Demographics	Colombia					Uganda				
	Hospital services	Community health	New medicine	Don’t know	p-value	Hospital services	Community health	New medicine	Don’t know	p-value
**Sex**					0.37					**0.004**
Male	180 (20.52)	579 (66.02)	24 (2.74)	94 (10.72)		270 (17.86)	582 (38.49)	127 (8.40)	533 (35.25)	
Female	166 (17.70)	652 (69.51)	28 (2.99)	92 (9.81)		50 (13.51)	120 (32.43)	39 (10.54)	161 (43.51)	
**Age (years)**					0.55					0.23
18–29	158 (18.37)	593 (68.95)	23 (2.67)	86 (10.00)		205 (16.00)	482 (37.63)	126 (9.84)	468 (36.53)	
30–49	127 (18.90)	448 (66.67)	19 (2.83)	78 (11.61)		104 (19.55)	195 (36.65)	34 (6.39)	199 (37.41)	
50+	61 (21.55)	190 (67.14)	10 (3.53)	22 (7.77)		11 (15.94)	25 (36.23)	6 (8.70)	27 (39.13)	
**Education**					**<0.001**					**<0.001**
None	2 (10.00)	10 (50.00)	5 (25.00)	3 (15.00)		42 (15.73)	46 (17.23)	23 (8.61)	156 (58.43)	
Primary	64 (27.47)	135 (57.94)	6 (2.58)	28 (12.02)		78 (15.63)	135 (27.05)	57 (11.42)	229 (45.89)	
Secondary	129 (20.28)	419 (65.88)	18 (2.83)	70 (11.01)		136 (18.11)	326 (43.41)	62 (8.26)	227 (30.23)	
Tertiary/higher	76 (16.34)	343 (73.76)	10 (2.15)	36 (7.74)		63 (17.40)	194 (53.59)	24 (6.63)	81 (22.38)	
Technical	75 (16.41)	320 (72.02)	13 (2.84)	49 (10.72)		-	-	-	-	
**Location**			** **		**0.003**					**0.017**
Urban	258 (19.55)	914 (69.24)	32 (2.42)	116 (8.79)		174 (18.67)	351 (37.66)	92 (9.87)	315 (33.80)	
Rural	88 (18.26)	308 (63.90)	20 (4.15)	66 (13.69)		145 (15.31)	350 (36.96)	74 (7.81)	378 (39.92)	
**Language**					NA					**<0.001** *
Spanish	346 (19.06)	1231 (67.82)	52 (2.87)	186 (10.25)						
Luganda	-	-	-	-		183 (18.01)	292 (28.85)	72 (7.11)	465 (45.95)	
Luo	-	-	-	-		29 (19.33)	60 (40.00)	14 (9.33)	47 (31.33)	
Runyakitara	-	-	-	-		51 (11.83)	181 (42.00)	44 (10.21)	155 (35.96)	
English	-	-	-	-		57 (19.72)	169 (58.48)	36 (12.46)	27 (9.34)	

Note: Data are n (%) for respondents who were defined as complete interviews.

* The Spearman correlation between language and education is <0.01.

In Uganda, overall, 37.30% of respondents correctly identified the survey purpose (95% CI: 35.14% - 39.51%) with no significant difference by study arm or age; however, a greater proportion of men correctly identified the survey purpose compared to women (38.49% and 32.43%, respectively (p = 0.004) **(Table 4, Fig 3B)**. Understanding also differed by location–urban vs rural (p<0.001). The proportion that correctly identified the survey purpose increased with educational attainment (17.23% none, 27.05% primary, 43.41% secondary, and 53.59% tertiary, p<0.001) **(Table 4, Fig 4B)**. There was also a significant difference (p<0.001) in the correct response based on the language chosen; whether Luganda (n = 292, 28.85%), Luo (n = 60, 40.00%), Runyakitara (n = 181, 42.00%), or English (n = 169, 58.48%) **(Table 4)**. A higher proportion of respondents (p = 0.014) gave the correct response after having completed a majority of the survey (41.98%, 95% CI: 36.43–47.73) than respondents who were asked about the survey purpose shortly after consent (32.22%, 95% CI: 27.17–37.73) **(Fig 3D)**. Survey understanding by study arms are also reported in **S5 Table**.

We reported the unadjusted and adjusted RR for the association of survey purpose understanding with arms and sociodemographic characteristics by country in **Table 5.** Overall, though rural residents had negative association with understanding in unadjusted analysis (unadjusted RR: 0.92, 95% CI: 0.85–0.99, p = 0.039), none of the factors, including survey arms were associated during adjusted analysis in Colombia (p>0.05). In Uganda, in adjusted analysis, people with primary (adjusted RR: 1.55, 95% CI: 1.14–2.08, p = 0.004), secondary (adjusted RR: 2.49, 95% CI: 1.89–3.29, p<0.001), and tertiary (adjusted RR: 3.16, 95% CI: 2.39–4.18, p<0.001) education level were more likely to understand than those without any formal education, however, survey arms were not significantly associated.

**Table 5:** Association of correct response with sociodemographic characteristics and study arms by country.

Variable	Colombia				Uganda			
	Unadjusted		Adjusted^1^		Unadjusted		Adjusted^1^	
	RR (95% CI)	P-value	RR (95% CI)	P-value	RR (95% CI)	P-value	RR (95% CI)	P-value
**Gender**								
Male	Ref.		Ref.		Ref.		Ref.	
Female	1-.05 (0.99,1.20)	0.11	1.03 (0.97,1.11)	0.25	0.84 (0.72, 0.99)	**0.036**	0.86 (0.74, 1.00)	0.056
**Age (in year)**								
18–29	Ref.		Ref.		Ref.		Ref.	
30–49	0.97 (0.90, 1.67)	0.34	0.99 (0.92, 1.06)	0.76	0.97 (0.85, 1.11)	0.70	1.03 (0.91, 1.17)	0.64
50+	0.69 (0.89, 1.07)	0.57	0.99 (0.90, 1.09)	0.79	0.96 (0.70, 1.32)	0.82	1.04 (0.76, 1.42)	0.81
**Education**								
None	Ref.		Ref.		Ref.		Ref.	
Primary	1.15 (0.74, 1.82)	0.52	1.13 (0.72, 1.78)	0.59	1.57 (1.16, 2.11)	**0.003**	1.55 (1.14, 2.08)	**0.004**
Secondary	1.31 (0.85, 2.04)	0.22	1.28 (0.82, 1.99)	0.28	2.52 (1.91, 3.31)	**<0.001**	2.49 (1.89, 3.29)	**<0.001**
Tertiary	1.48 (0.95, 2.29)	0.08	1.41 (0.91, 2.20)	0.13	3.11 (2.35, 4.11)	**<0.001**	3.16 (2.39, 4.18)	**<0.001**
Technical	1.40 (0.90, 2.17)	0.14	1.41 (0.91, 2.10)	0.18	NA		NA	
**Location**								
Urban	Ref.		Ref.		Ref.		Ref.	
Rural	0.92 (0.85, 0.99)	**0.039**	0.96 (0.89, 1.04)	0.30	0.98 (0.87, 1.10)	0.75	1.09 (0.97, 1.21)	0.15
**Survey Arm**								
SI, OI OO	Ref.		Ref.		Ref.		Ref.	
MI, OI OO	0.99 (0.89, 1.09)	0.79	1.00 (0.91,1.11)	0.97	1.04 (0.86, 1.26)	0.72	1.03 (0.86, 1.24)	0.76
MI, OI	0.97 (0.88, 1.08)	0.60	0.98 (0.89, 1.09)	0.72	1.05 (0.87, 1.27)	0.63	1.03 (0.85, 1.23)	0.78
MI, OO	1.00 (0.90, 1.10)	0.97	1.00 (0.90, 1.10)	0.97	1.09 (0.91, 1.32)	0.35	1.08 (0.90, 1.29)	0.40
MI, Implied	1.05 (0.96, 1.16)	0.29	1.06 (0.96, 1.16)	0.26	1.16 (0.97, 1.40)	0.11	1.17 (0.98, 1.40)	0.074

**Abbreviations:** CI: Confidence interval, MI: Modified Intro, NA: Not available, OI: Opt-in, OO: Opt-out, RR: Risk ratio, SI: Standard Intro

1. *Adjusted for all variables in the columns*

## Discussion

This study comparatively evaluated modifications to consent disclosure and authorization modalities for an IVR deployed in Colombia and Uganda to explore relationships between the consent approach and survey participation and understanding outcomes. We also sought to identify whether timing of measuring participant understanding of the purpose of the survey (i.e., measurement immediately after consent vs. near the end of the IVR NCD survey) was associated with higher or lower levels of understanding. Forms of information disclosure (i.e., standard and modified) and modes of authorization (i.e., opt-in, opt-out, combined opt-in/opt-out, or implied) had significant effects on primary outcomes of cooperation and response rates, with no effect on participants’ understanding of the purpose of the survey. In both countries, participant understanding was substantially higher when measured later in the survey, as compared to immediately following disclosure of information at consent. This study contributes to the limited available evidence on consent procedures when mobile devices are used to facilitate disease research and surveillance in LMICs.

Our study found associations between aspects of the consent approach and both survey disposition and respondent understanding of the purpose of the survey, with differences by country context. In Colombia, there was a clear indication against the opt-out form of authorization, as evidenced by the significantly higher refusal rate and the significantly lower response and cooperation rates. Recently introduced Colombian data protection regulations are stringent in requiring affirmative forms of authorization for data access and use [28]. These regulations likely reflect pre-existing preferences and have the added effect of raising awareness of and shaping attitudes against default forms of authorization.

In Uganda, compound modes of authorization (i.e., those that included both opt-in and opt-out modes) were associated with higher refusal rates and lower cooperation rates, raising the possibility that these modes of authorization may pose difficulties for respondents. It is unclear whether higher levels of performance across singular opt-in, opt-out, and implied forms of authorization align with stated preferences. It has been suggested elsewhere that an opt-in form of authorization for MPS might be preferable to the Ugandan population [9]. Discrepancies between behaviors and stated preferences have been noted in related areas, including in relation to digital privacy and what has been described as the “privacy paradox” [29]. Additional research is needed to further understand the observed differences in both countries in respondent disposition, as it relates to the mode of authorization.

While some information can be more difficult to convey than the purpose of a survey–for instance the risks and benefits, or the idea of random assignment–communicating the purpose of a survey is still known to be challenging [30]. Compared to Colombia, where nearly two-thirds of respondents correctly identified the survey to be related to community or population health, in Uganda, only slightly more than one-third of respondents did so. In both countries, a higher level of respondent education was associated with correct identification of the survey purpose, which is consistent with prior consent understanding studies outside the MPS context. However, Colombia generally has higher levels of educational attainment, which was reflected in our study data; in Colombia, approximately 14% reported completing no more than primary education, compared to 40% in Uganda. In addition, we observed a significant difference in the correct response based on the language chosen. The association between level of education and language, especially for English, may account for the effects of education on consent understanding.

It is particularly noteworthy that despite the consent modules in both countries explicitly mentioning the purpose of the survey, those who were asked about its purpose immediately after the consent process were less likely to correctly identify that the survey sought to advance community or population health as compared to those who were asked about the survey’s purpose at the end of the questionnaire. Several studies have similarly shown that participants may not fully understand the purpose of a research activity immediately following consent [30]. Further, studies involving consent for research have found that the timing of measurement of understanding is relevant to interpretation of findings, with an emphasis on the potential for recall bias in measurement [31]. While there is a generally accepted notion of consent being a "process" (and not a one-time event), there is limited evidence demonstrating an evolution of consent understanding throughout the research process, as we have shown here.

Shortcomings in understanding can be attributable to different factors, and it can be difficult to infer which of these might be most salient for a given individual or context. The overall difference in understanding noted between countries in our study could be influenced by the level of education of the study population as well as the language of survey administration. The sole survey language used in Colombia was Spanish, with near-universal coverage across the population, while in Uganda the survey was administered in four of the approximately 43 languages spoken [32]. This may have the effect of some respondents choosing familiar but non-native languages, impacting understanding of the survey’ purpose or its questions.

Consent is not a static or singular construct—norms and behaviors related to consent may shift with changes in how societies, governments and health system actors collect and utilize information. Many acknowledge that consent expectations vary depending on the nature of the activity being consented to [33]. For a simple, low-risk automated MPS that presents opportunities for meaningful public health benefit, there is a goal of developing a consent process that is effective and efficient. Future research that measures other dimensions of consent understanding for MPS, such as survey procedures, risks, benefits, and perceptions of voluntariness in participation would be valuable. Further research to explore whether certain respondents experience technical challenges when effectuating their participation preferences using different modes of authorization (i.e., opt-in vs opt-out) would also be valuable. Finally, it is important to consider opportunities to develop more interactive consent processes for automated surveys, such as by introducing the option for conversation with a live operator who can answers questions.

This study has several limitations. Though we used a randomized trial design and had a large sample size, the sample was not nationally representative and included a higher proportion of people who were younger, more highly educated, or urban residents. Moreover, cross-country comparisons were somewhat difficult to make for linguistic reasons (despite best efforts, some concepts may not translate identically across multiple languages), and due to different software platforms being used to administer the MPS in each country, given local availability. While there was no meaningful difference in experience of the survey from a respondent perspective, there were differences in how the two survey systems handled randomization of survey modules. In Uganda, module randomization was automated, whereas in Colombia we had to manually allocate randomly generated blocks of phone numbers to receive different module order assignments. Finally, due to constraints on survey length (it is well known that many respondents drop off IVR surveys if they are too long) we were only able to introduce a one-item measure of understanding. A more robust study of consent for MPS might examine additional elements of understanding to establish a more comprehensive understanding score. Given the novelty of this study, we elected to take an incremental approach to consent understanding measurement.

## Conclusion

Mobile phones demonstrate increasing promise as a means of improving public health data collection, including through MPS. Additional attention is needed to optimizing consent processes for such technology uses. This study contributes to the limited available evidence regarding remote consent procedures for automated MPS, identifying how small variations to the mode of authorization can have an effect on respondent disposition. Future studies should develop and trial additional interventions to enhance consent for automated public health surveys, and measure other dimensions of participant engagement and understanding. Preferences for different modes of authorization can also be further examined in different country- and data-contexts. Research of this nature can not only contribute improvements to survey methodology, but also enhance the ethical quality of automated MPS.

## Supporting information

S1 Checklist Inclusivity in global research. (DOCX)

S1 Table Standard and modified survey introductions (English language versions). (DOCX)

S2 Table Back-translated measures of understanding of the purpose of the survey. (DOCX)

S3 Table AAPOR definitions and equations. (DOCX)

S4 Table Disposition codes by study arm in Colombia and Uganda. (DOCX)

S5 Table Survey understanding by study arm and timing of measurement in Colombia and Uganda. (DOCX)
