# Supplementary material for: Remote consent approaches for mobile phone surveys of non-communicable disease risk factors in Colombia and Uganda: A randomized study
Source: PLoS One. 2022 Dec 21;17(12):e0279236. doi: 10.1371/journal.pone.0279236 (PMC9770397; doi:10.1371/journal.pone.0279236)
Supplement: S1 Table — (DOCX) [file pone.0279236.s002.docx]

**S1 Table. Standard and modified survey introductions (English language versions)**

|  | **Standard** | **Modified** |
| --- | --- | --- |
| **Uganda** | Hello, we are conducting a health survey from Makerere University. This interview will take no more than 15 minutes of your time. Any information you share will be kept confidential and private. To answer each question, I will ask you to press a number - for example, 1 for YES, and 3 for NO, or to answer a question with a number - like 12 or 22. You can hear a question again at any time by pressing the STAR button - located to the left of the ZERO at the bottom of your phone. As a token of appreciation, we will send you 4000 Uganda shillings of airtime for completing the survey. | This is a routine public health survey being conducted by Makerere University. The survey will collect information about your health and some common diseases. This survey is not about treatment of diseases. It is only to understand more about the health of our country’s people. This survey will take no more than 15 minutes of your time. While we hope you will be able to answer the questions, you do not need to complete the survey if you do not want to.  As a token of appreciation, we will send you 4000 Ugandan shillings of airtime for completing the survey. We are using your phone number to contact you, but the information you share will not be connected to you personally. To answer each question, I will ask you to press a number. You can hear a question again at any time by pressing the STAR button - located at the bottom left of your phone. For most of the questions, if you do not want to answer, you can press the ZERO key, unless otherwise indicated. |
| **Colombia** | Hello, Javeriana University of Bogota is conducting a national survey to get a better understanding of the health habits that affect the Colombian population, with the purpose of designing better health programs for your and the society’s benefit. Your participation is of great importance for this purpose and the survey will not take longer than 10 minutes. Your identity will remain anonymous and responses confidential. You will not be asked to provide any personal or contact information. The survey will not carry any cost for you. Your phone number was generated automatically, and therefore it was not obtained through a database. To answer the questions, use the keyboard on your phone. You can hear the question again at any time by pressing STAR located in the bottom left part of your phone. If you don’t want to answer a question press 0 (zero.) to skip it. | Hello, Javeriana University of Bogota is conducting a national survey to get a better understanding of the health habits that affect the Colombian population, with the purpose of designing better health programs for your and the society’s benefit. Your participation is of great importance for this purpose and the survey will not take longer than 10 minutes. We hope you can answer the survey, but you are not obliged to do so. Your identity will remain anonymous and responses confidential. You will not be asked to provide any personal or contact information. The survey will not carry any cost for you. Your phone number was generated automatically, and therefore it was not obtained through a database. To answer the questions, use the keyboard on your phone. You can hear the question again at any time by pressing STAR located in the bottom left part of your phone. If you don’t want to answer a question press 0 (zero.) to skip it. |
