# Supplementary material for: Remote consent approaches for mobile phone surveys of non-communicable disease risk factors in Colombia and Uganda: A randomized study
Source: PLoS One. 2022 Dec 21;17(12):e0279236. doi: 10.1371/journal.pone.0279236 (PMC9770397; doi:10.1371/journal.pone.0279236)
Supplement: S2 Table — (DOCX) [file pone.0279236.s003.docx]

**S2 Table. Back-translated measures of understanding of the purpose of the survey**

| **Uganda** | **Colombia** |
| --- | --- |
| **Why did we contact you today to complete this survey? What do you think we are trying to find out?**  Press 1 if you think we are trying to improve understanding of hospital services. Press 2 if you think we are trying to improve understanding of community health. Press 3 if you think we are trying to develop a new medicine. Press 7 if you do not know the reason why we contacted you today. | **What do you think was the purpose of this survey?**  Get knowledge and data about the performance of the health services, press 1. Get knowledge and data about the health conditions of the population, press 2. Develop a new medicine, press 3. If you don’t know the reason why we contacted you, press 7. |
