# Supplementary material for: Remote consent approaches for mobile phone surveys of non-communicable disease risk factors in Colombia and Uganda: A randomized study
Source: PLoS One. 2022 Dec 21;17(12):e0279236. doi: 10.1371/journal.pone.0279236 (PMC9770397; doi:10.1371/journal.pone.0279236)
Supplement: S3 Table — (DOCX) [file pone.0279236.s004.docx]

**S3 Table. AAPOR definitions and equations**

| **Disposition Codes** | | **Definition/Equations** |
| --- | --- | --- |
| **AAPOR Definitions** | Complete Interviews (I) | Participants who answered at least 5 of the 7 modules. |
|  | Partial Interviews (P) | Participants who answered 2,3, or 4 modules. |
|  | Refusals (R) | Participants who either did not press a button on their mobile phone to indicate consent, refused consent, or who hung-up at the consent question. |
|  | Break-offs (R) | Participants who consented but did not complete a module other than demographics |
|  | Unknown (U) | Participants who selected a survey language but did not answer the age question. |
|  | Estimated Unknown (eU) | Estimated proportion of unknown cases that were age eligible. |
|  | Ineligible on age | Participant who indicated an age less than 18 years of age. |
| **AAPOR Equations** | Contact Rate #2 | $\frac{(I+P+R+O)}{I+P+R+O+e(UH+UO)}$ |
|  | Response Rate #4 | $\frac{(I+P)}{I+P+R+O+e(UH+UO)}$ |
|  | Refusal Rate #2 | $\frac{(R)}{I+P+R+O+e(UH+UO)}$ |
|  | Cooperation Rate #1 | $\frac{(I)}{I+P+R+O}$ |
