# Supplementary material for: Remote consent approaches for mobile phone surveys of non-communicable disease risk factors in Colombia and Uganda: A randomized study
Source: PLoS One. 2022 Dec 21;17(12):e0279236. doi: 10.1371/journal.pone.0279236 (PMC9770397; doi:10.1371/journal.pone.0279236)
Supplement: S4 Table — (DOCX) [file pone.0279236.s005.docx]

**S4 Table. Disposition codes by study arm in Colombia and Uganda**

|  | **Colombia** | | | | | **Uganda** | | | | |
| --- | --- | --- | --- | --- | --- | --- | --- | --- | --- | --- |
|  | **Standard Intro, Opt-in Opt-out** | **Modified Intro, Opt-in Opt-out** | **Modified Intro, Opt-in** | **Modified Intro, Opt-out** | **Modified Intro, Implied** | **Standard Intro, Opt-in Opt-out** | **Modified Intro, Opt-in Opt-out** | **Modified Intro, Opt-in** | **Modified Intro, Opt-out** | **Modified Intro, Implied** |
| **Complete Interview (I)** | 414 | 382 | 388 | 364 | 341 | 376 | 377 | 378 | 380 | 379 |
| **Partial Interview (P)** | 124 | 109 | 136 | 90 | 121 | 97 | 99 | 73 | 83 | 83 |
| **Refusal** **(R)** |  |  |  |  |  |  |  |  |  |  |
| Breaks-off | 102 | 95 | 136 | 115 | 236 | 59 | 48 | 45 | 74 | 55 |
| Refusal | 155 | 155 | 88 | 361 | 0 | 81 | 67 | 40 | 15 | 0 |
| ***e* Unknown *e*(U)*** | 35,832 | 39,992 | 39,828 | 50,393 | 37,368 | 23,050 | 23,126 | 23,038 | 23,093 | 23,049 |
| **Ineligible** |  |  |  |  |  |  |  |  |  |  |
| Underage | 109 | 99 | 79 | 112 | 77 | 50 | 57 | 43 | 58 | 54 |
| Average time per call | 9 min 11 s | 9 min 4 s | 9 min 16 s | 9 min 33 s | 9 min 10 s | 10 min 27 s | 10 min 42 s | 10 min 58 s | 10 min 50s | 10 min 50s |

*estimated Unknown numbers were obtained by (1) dividing the total number of age-ineligible with total number of unknown calls, (2) subtracting the proportion obtained in Step 1 from 100%, and (3) Multiplying proportion obtained in Step 2 with unknown number of each arm
