# Supplementary material for: Remote consent approaches for mobile phone surveys of non-communicable disease risk factors in Colombia and Uganda: A randomized study
Source: PLoS One. 2022 Dec 21;17(12):e0279236. doi: 10.1371/journal.pone.0279236 (PMC9770397; doi:10.1371/journal.pone.0279236)
Supplement: S5 Table — (DOCX) [file pone.0279236.s006.docx]

**S5 Table. Survey understanding by study arm and timing of measurement in Colombia and Uganda**

| **Responses** | **By Study Arm** | | | | | **By Timing of Measurement** | | **Overall** |
| --- | --- | --- | --- | --- | --- | --- | --- | --- |
|  | **Standard Intro,**  **Opt-in Opt-out** | **Modified Intro,**  **Opt-in Opt-out** | **Modified Intro,**  **Opt-in** | **Modified Intro,**  **Opt-out** | **Modified Intro,**  **Implied** | **First** | **Last** |  |
| **Colombia** | | | | | | | | |
| **Hospital services** | 17.75  (14.30-21.82) | 23.56  (19.48-28.20) | 19.30  (15.60-23.63) | 19.20  (15.39-23.68) | 15.24  (11.74-19.56) | 27.43  (24.47-30.60) | 12.28  (10.38-14.46) | 19.06  (17.32-20.93) |
| **Community health*** | 67.75  (63.00-72.16) | 66.85  (61.85-71.50) | 65.95  (60.98-70.59) | 67.62  (62.52-72.33) | 71.34  (66.20-75.99) | 54.24  (50.80-57.65) | 78.84  (76.20-81.26) | 67.82  (65.63-69.94) |
| **New medicine** | 4.50  (2.85-7.03) | 1.64  (0.74-3.62) | 2.14  (1.07-4.23) | 4.30  (2.60-7.01) | 1.52  (0.63-3.62) | 3.57  (2.49-5.09) | 2.30  (1.53-3.43) | 2.87  (2.19-3.74) |
| **Don't know** | 10.00  (7.42-13.35) | 7.95  (5.57-11.21) | 12.60  (9.59-16.38) | 8.88  (6.31-12.36) | 11.89  (8.80-15.87) | 14.76  (12.48-17.37) | 6.59  (5.21-8.30) | 10.25  (8.93-11.73) |
| **Uganda** | | | | | | | | |
| **Hospital services** | 16.53  (13.10-20.65) | 18.88  (15.23-23.17) | 16.71  (13.27-20.83) | 16.45  (13.03-20.54) | 16.45  (13.03-20.54) | 18.27  (14.29-23.07) | 17.06  (13.16-21.83) | 17.00  (15.37-18.77) |
| **Community health*** | 34.93  (30.27-39.91) | 36.17  (31.46-41.16) | 36.60  (31.88-41.60) | 38.20  (33.42-43.22) | 40.58  (35.73-45.63) | 32.23  (27.17-37.74) | 41.98  (36.44-47.73) | 37.30  (35.14-39.51) |
| **New medicine** | 9.33  (6.77-12.73) | 7.71  (5.41-10.89) | 6.90  (4.73-9.94) | 10.88  (8.10-14.45) | 9.28  (6.74-12.66) | 5.65  (3.53-8.91) | 7.85 (5.26 - 11.55) | 8.82  (7.62-10.19) |
| **Don't know** | 39.20  (34.37-44.25) | 37.23  (32.48-42.25) | 39.79  (34.96-44.83) | 34.48  (29.85-39.44) | 33.69  (29.08-38.62) | 43.85  (38.33-49.54) | 33.11  (27.94-38.72) | 36.88  (34.72-39.08) |

**Note:** Data are % and CI for respondents who were defined as complete interviews.

*Correct response
